# Knockdown of BAP31 Overcomes Hepatocellular Carcinoma Doxorubicin Resistance through Downregulation of Survivin

**DOI:** 10.3390/ijms24087622

**Published:** 2023-04-21

**Authors:** Jingjing Liu, Qi Zhang, Changli Wang, Jiaying Yang, Sheng Yang, Tianyi Wang, Bing Wang

**Affiliations:** Institute of Biochemistry and Molecular Biology, College of Life and Health Sciences, Northeastern University, Shenyang 110819, China

**Keywords:** BAP31, doxorubicin, chemoresistance, apoptosis, survivin

## Abstract

The expression of B-cell receptor associated protein 31 (BAP31) is increased in many tumor types, and it is reported to participate in proliferation, migration, and apoptosis. However, the relationship between BAP31 and chemoresistance is uncertain. This study investigated the role of BAP31 in regulating the doxorubicin (Dox) resistance of hepatocellular carcinoma (HCC). The expression of proteins was assessed by Western blotting. The correlation between BAP31 expression and Dox resistance was examined by MTT and colony formation assays. Apoptosis was analyzed by flow cytometry and TdT-mediated dUTP nick end labeling assays. Western blot and immunofluorescence analyses were performed in the knockdown cell lines to explore the possible mechanisms. In this study, BAP31 was strongly expressed, and knockdown of BAP31 increased Dox chemosensitivity in cancer cells. Furthermore, the expression of BAP31 was higher in the Dox-resistant HCC cells than that in their parental cells; knockdown of BAP31 reduced the half maximal inhibitory concentration value and overcame Dox resistance in Dox-resistant HCC cells. In HCC cells, knockdown of BAP31 increased Dox-induced apoptosis and enhanced Dox chemosensitivity in vitro and in vivo. The potential mechanism by which BAP31 increased Dox-induced apoptosis is that BAP31 inhibited survivin expression by promoting FoxO1 nucleus–cytoplasm translocation. Knockdown of BAP31 and survivin had a synergistic effect on Dox chemosensitivity by enhancing the apoptosis of HCC cells. These findings reveal that BAP31 knockdown enhances Dox chemosensitivity through the downregulation of survivin, suggesting that BAP31 is a potential therapeutic target for improving the treatment response of HCC with resistance to Dox.

## 1. Introduction

Chemoresistance is a complex phenomenon regulated by various mechanisms, and it is a main hindrance to treatment in most human tumors [1,2]. Drug resistance may arise intrinsically from host factors or may be acquired via genetic or epigenetic alterations of cancer cells [3]. Drug resistance confers the ability of cancer cells to regulate rates of drug efflux, DNA damage repair processes, and signaling pathways affecting cell survival and death [4,5]. Doxorubicin (Dox) is invoked as one of the most effective agents in clinical treatment. As the first-line proliferation inhibitor drug to treat cancers, Dox is widely used in postoperative adjuvant chemotherapy for hepatocellular carcinoma (HCC) [6], breast cancer [7], and pancreatic cancer [8]. Dox remains the major chemotherapeutic agent used to treat a wide variety of solid malignancies [9]. Unfortunately, chemotherapeutic resistance to Dox has emerged as one of the essential reasons for treatment failure and death associated with cancer [10]. Multiple mechanisms have been reported to underlie Dox resistance, including drug target mutation, apoptosis disorder, signaling alteration, reprogramming of metabolism, and efflux of drugs [11,12]. Thus, revealing the underlying mechanism and discovering novel therapeutic targets are necessary to improve patient treatment outcomes.

B-cell receptor associated protein 31 (BAP31) is an integral polytopic endoplasmic reticulum membrane protein [13]. High expression of BAP31 has been demonstrated in a variety of cancers, such as hepatocellular carcinoma [14,15], lung cancer [16], and cervical cancer [17]. BAP31 could act as an oncogene in cancer cells, promoting survival and growth through a series of functions and predicting poor prognosis. Meanwhile, BAP31 involves various physiological and pathological processes such as immunity and carcinogenesis [18,19]. It is reported that BAP31 depletion inhibits cell invasion and migration by modulating cytoskeleton assemblage in cervical cancer [17]. In ovarian cancer, BAP31 modulates migration and invasion via the epithelial–mesenchymal transition process [20]. In our previous study, BAP31 regulated Wnt signaling to modulate cell migration in lung cancer [16]. In addition, an intrabody induces gastric cancer cell death by regulating p27 proteasome degradation [21]. Despite extensive research on BAP31 in cancer, the role of BAP31 in regulating drug resistance is still largely unknown. A comprehensive understanding of the mechanisms of BAP31 in regulating chemoresistance in cancer cells would enhance the effectiveness of chemotherapy agents and improve prognoses.

The mechanisms of chemoresistance of cancer cells are complex, including increased drug efflux, changes in anticancer drug targets, signaling pathways affecting cell survival and death, etc. [22]. Evasion of cell apoptosis is recognized as one of the mechanisms responsible for drug resistance [23,24]. Survivin is a member of the inhibitor of apoptosis protein family, which has been intensively studied, as it is over-expressed in most human cancer cells, rendering them resistant to cytotoxic chemotherapy [25,26]. The research demonstrated that survivin participates in the resistance of gastric cancer to cisplatin [27]. Survivin also promotes piperlongumine resistance in ovarian cancer [28]. Furthermore, it has been reported that BAP31 can transcriptionally downregulate the expression of specific anti-apoptotic factors, including Bcl-2, in certain cell types [29]. By analyzing the relationship between BAP31 and 84 types of tumor-associated antigens using a Proteome Profiler Human XL Oncology Array, we found that overexpression of BAP31 increased the expression of survivin. Therefore, we investigated whether BAP31 affects Dox chemosensitivity by regulating survivin.

In this study, we demonstrated that the high expression of BAP31 is related to Dox resistance in HCC cells. Knockdown of BAP31 reduces survivin expression by enhancing the nucleus–cytoplasm translocation of FoxO1, which increases HCC chemosensitivity to Dox. Thus, targeting BAP31 may be a strategy to improve the antitumor effect of Dox in HCC.

## 2. Results

### 2.1. BAP31 Is Increased in Cancer Cells and Associated with Chemosensitivity to Dox

**Figure 1:**
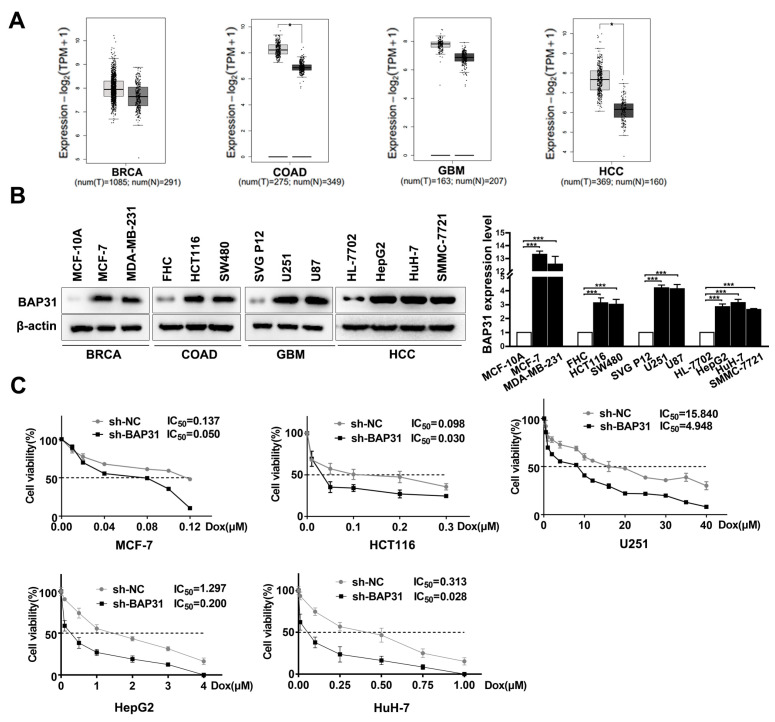
B-cell receptor associated protein 31 (BAP31) is increased in cancer cells and associated with chemosensitivity to doxorubicin (Dox). (**A**) A large-scale dataset analysis using GEPIA confirmed the expression of BAP31 in the cancer tissues compared with that in normal tissues. (**B**) Western blot was used to detect the expression of BAP31 in indicated cancer and normal cells. (**C**) 3-(4,5-dimethyl-2-thiazolyl)-2,5-diphenyl-2-H-tetrazolium bromide (MTT) assay was used to assess the half maximal inhibitory concentration (IC_50_) value of the indicated cells treated with Dox. Data are represented as the mean ± SD of three independent experiments. β-actin was used as the loading control. * *p* < 0.05, *** *p* < 0.001.

Previous studies have demonstrated that BAP31 exhibits a strong association with tumorigenesis in most types of cancers. A large-scale dataset analysis using GEPIA confirmed that BAP31 was overexpressed in cancer tissues compared to normal tissues (Figure 1A). To ensure these observations, we detected BAP31 expression in normal and cancer cells. Western blot results showed that the expression of BAP31 was markedly increased in breast invasive carcinoma (BRCA), colon adenocarcinoma (COAD), glioblastoma multiforme (GBM), and HCC cells compared with normal cells (Figure 1B). Together, these data suggested that BAP31 is highly expressed in cancer cells. To test whether BAP31 could influence the chemosensitivity of cancer cells to Dox, we established stable BAP31 knockdown cell lines. Western blot and quantitative real-time polymerase chain reaction (qRT-PCR) assays showed that BAP31 was significantly reduced in sh-BAP31 cancer cells (Figure S1A,B). The cells were then treated with increasing concentrations of Dox for 48 h to assess cell viability by 3-(4,5-dimethyl-2-thiazolyl)-2,5-diphenyl-2-H-tetrazolium bromide (MTT) and colony formation assay. MTT assay showed that knockdown of BAP31 decreased the half maximal inhibitory concentration (IC_50_) value and increased the chemosensitivity of cancer cells to Dox (Figure 1C). Consistently, the number of colonies formed by sh-NC cells was higher than that formed by sh-BAP31 cells treated with Dox; knockdown of BAP31 dramatically decreased the colony formation ability of cancer cells (Figure S1C). These data indicated that BAP31 participates in chemosensitivity to Dox in cancer cells.

### 2.2. Knockdown of BAP31 Reduces Dox Resistance in HCC/Dox Cells

**Figure 2:**
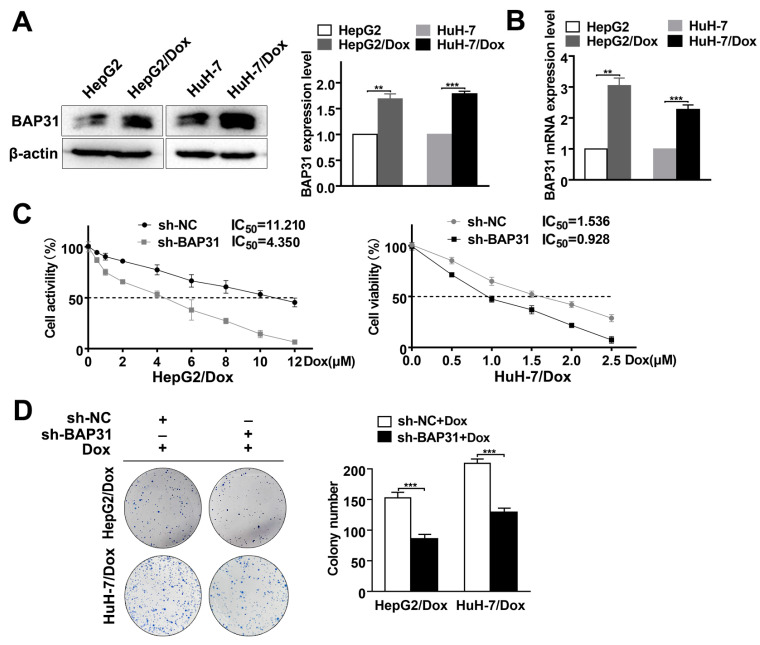
Knockdown of BAP31 reduces Dox resistance in hepatocellular carcinoma (HCC)/Dox cells. (**A**,**B**) Western blot and quantitative real-time polymerase chain reaction (qRT-PCR) assays were used to detect the expression of BAP31 in HCC and HCC/Dox cells. (**C**) MTT assay was used to measure the IC_50_ value of HCC/Dox cells with or without BAP31 knockdown under different concentrations of Dox. (**D**) The colony formation assay was used to measure the colony formation ability of HCC/Dox cells with or without BAP31 knockdown under the treatment of Dox. Data are represented as the mean ± SD of three independent experiments. β-actin was used as the loading control. ** *p* < 0.01, *** *p* < 0.001.

We established Dox-resistant cell lines (HepG2/Dox, HuH-7/Dox) from HepG2 and HuH-7 by long-term culture with low/increasing doses of Dox. Next, we measured the IC_50_ value of the HepG2/Dox and HuH-7/Dox cells to confirm Dox resistance (Figure S2A). These cells were highly drug-resistant and could be used in subsequent experiments. To detect the expression of BAP31, Western blot and qRT-PCR assays were conducted. In HepG2/Dox and HuH-7/Dox cells, BAP31 expression was higher compared with that in HepG2 and HuH-7 cells (Figure 2A,B).

To test the effect of BAP31 on Dox resistance, we established stable BAP31 knockdown cell lines in HCC/Dox cells. Western blot and qRT-PCR assays showed that the expression of BAP31 was significantly reduced (Figure S2B,C). Next, using the MTT assay, we found that the IC_50_ value of sh-BAP31 cells was markedly lower than that of sh-NC HCC/Dox cells (Figure 2C). In addition, the results of the colony formation experiment indicated that cell colony formation of sh-BAP31 HCC/Dox cells was significantly reduced compared with sh-NC HCC/Dox cells (Figure 2D). Together, these data confirmed that BAP31 knockdown reduces Dox resistance in HCC/Dox cells.

### 2.3. Knockdown of BAP31 Enhances Dox-Induced Apoptosis in HCC Cells

**Figure 3:**
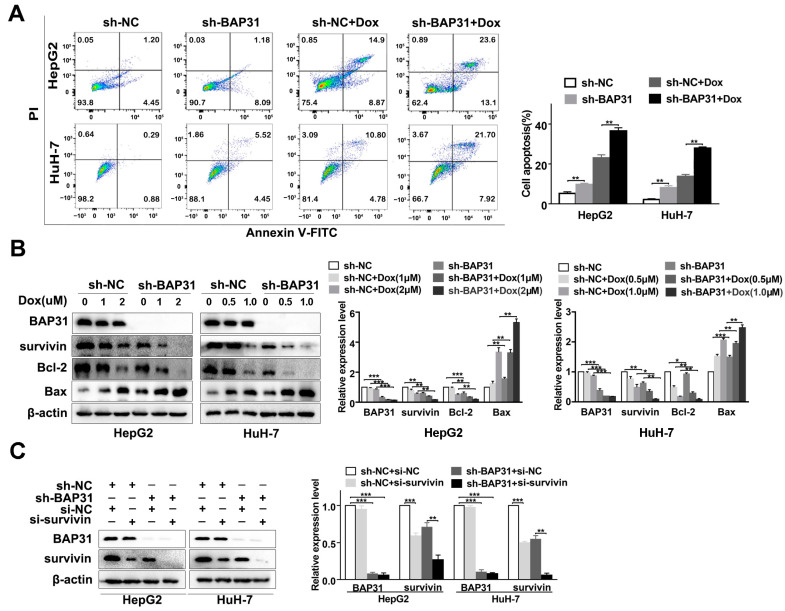
Knockdown of BAP31 increases Dox-induced apoptosis in HCC cells. (**A**) Annexin V-FITC and PI staining showing apoptosis in indicated cancer cells treated with or without Dox. (**B**) Western blot was used to detect the expression of BAP31, survivin, Bcl-2, and Bax in indicated cancer cells treated with or without Dox. (**C**) Western blot was used to detect the expression of BAP31 and survivin in indicated cancer cells transfected with or without si-survivin. Data are represented as the mean ± SD of three independent experiments. β-actin was used as the loading control. * *p* < 0.05, ** *p* < 0.01, *** *p* < 0.001.

The induction of apoptosis is the primary mechanism used by most chemotherapeutic drugs to eliminate cancer cells. To address the potential role of BAP31 in Dox-induced apoptosis of HCC cells, we assessed apoptosis by performing flow cytometry and a TdT-mediated dUTP nick end labeling (TUNEL) assay. The results of the flow cytometry revealed that knockdown of BAP31 enhanced apoptosis and led to a considerable increase in Dox-induced apoptosis of HepG2 and HuH-7 cells (Figure 3A). A TUNEL assay was also performed to validate the results (Figure S3A). These results revealed that knockdown of BAP31 enhances apoptosis and enhances Dox-induced apoptosis in HCC cells.

Our previous study analyzed the relationship between BAP31 and 84 types of tumor-associated antigens using a Proteome Profiler Human XL Oncology Array. We discovered that BAP31 was positively correlated with survivin (Figure S3B). To further explore the effect of BAP31 on Dox-induced apoptosis, Western blot analyzed the expression of molecules that play essential roles in cell apoptosis and resistance, including survivin, Bcl-2, and Bax. As shown in Figure 3B, under Dox treatment, knockdown of BAP31 upregulated the expression of Bax and downregulated the expression of survivin and Bcl-2. These results suggested that knockdown of BAP31 may increase Dox chemosensitivity via promoting the apoptosis of HepG2 and HuH-7 cells. To substantiate the putative functional link between BAP31 and survivin, HepG2 and HuH-7 cells were treated with si-RNA (si-survivin) to silence survivin expression and exogenous survivin (survivin-HA) to increase survivin expression (Figure 3C and Figure S3C). The results showed that altering the expression of survivin did not affect the expression of BAP31 in HepG2 and HuH-7 cells. In summary, knockdown of BAP31 increases Dox-induced apoptosis in HCC cells, which may be related to the Dox chemosensitivity in HCC cells.

### 2.4. BAP31 Regulates Survivin through the Nucleus-Cytoplasm Translocation of FoxO1

**Figure 4:**
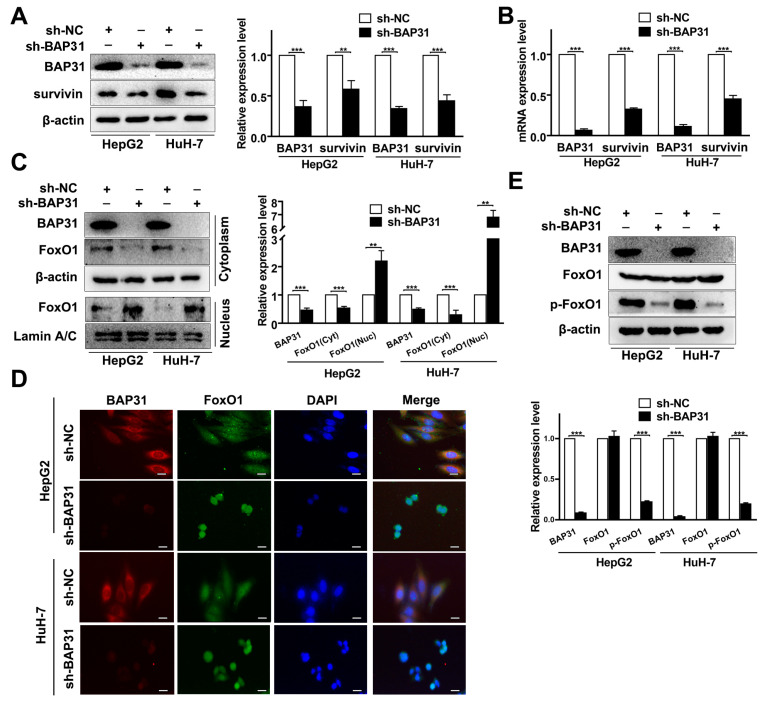
BAP31 regulates survivin through the nucleus–cytoplasm translocation of FoxO1. (**A**,**B**) Western blot and qRT-PCR assays were used to detect the expression of BAP31 and survivin in indicated cancer cells. (**C**) The cytoplasm and nucleus fractions of FoxO1 were analyzed by Western blot. (**D**) Distribution of BAP31 (red) and FoxO1 (green) as determined by immunofluorescence analysis (scale bars: 50 µm). Nuclei were stained with DAPI (blue). (**E**) Western blot was used to detect the expression of FoxO1 and p-FoxO1 in indicated cancer cells. Data are represented as the mean ± SD of three independent experiments. β-actin and lamin A/C were used as the loading control. ** *p* < 0.01, *** *p* < 0.001.

To explore the correlation between BAP31 and survivin, Western blot and qRT-PCR assays were performed. The results showed that survivin protein and mRNA expression decreased when BAP31 expression was downregulated, and their expression was positively correlated (Figure 4A,B). After that, we performed an immunoprecipitation assay and found that BAP31 did not interact with survivin in HepG2 and HuH-7 cells (Figure S3D). Considering that survivin is a target gene of FoxO1, we speculated that BAP31 affects the transcription of survivin by mediating FoxO1. We measured the effect of BAP31 on the nucleus–cytoplasm translocation of FoxO1. The Western blot and immunofluorescence analysis results revealed that the translocation of FoxO1 from the cytoplasm to the nucleus significantly increased when BAP31 was knocked down in HepG2 and HuH-7 cells (Figure 4C,D). Meanwhile, knockdown of BAP31 reduced the phosphorylation of FoxO1, which decreased FoxO1 in the cytoplasm of HepG2 and HuH-7 cells (Figure 4E). These results demonstrated that knockdown of BAP31 reduces survivin by enhancing the nucleus–cytoplasm translocation of FoxO1.

### 2.5. BAP31 and Survivin Have a Synergistic Effect on Chemosensitivity of Dox in HCC Cells

**Figure 5:**
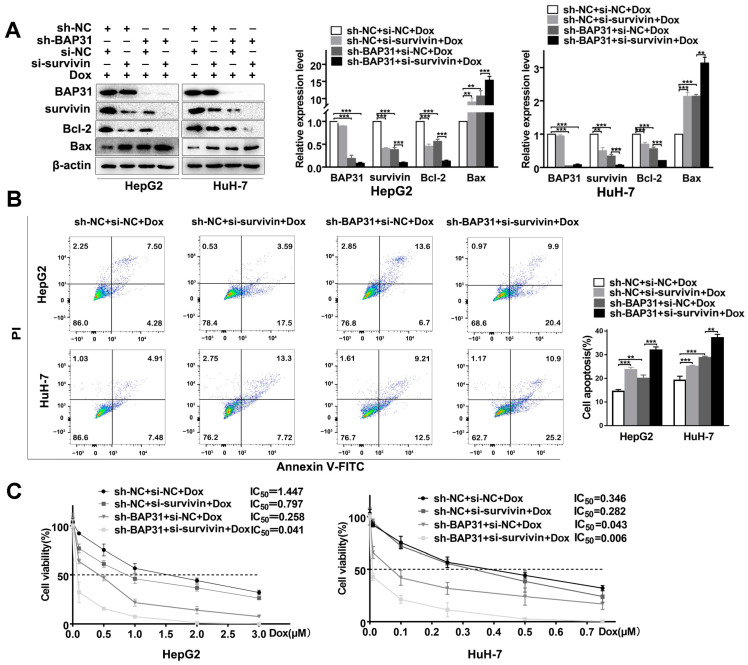
BAP31 and survivin have a synergistic effect on chemosensitivity of Dox in HCC cells. (**A**) Western blot was used to detect the expression of BAP31, survivin, Bcl-2, and Bax in indicated cancer cells transfected with or without si-survivin under the treatment with Dox. (**B**) Annexin V-FITC and PI staining showing apoptosis in indicated cancer cells transfected with or without si-survivin under the treatment with Dox. (**C**) MTT assay was used to measure the IC_50_ value in indicated cancer cells transfected with or without si-survivin under the treatment with Dox. Data are represented as the mean ± SD of three independent experiments. ** *p* < 0.01, *** *p* < 0.001.

Given the functional link between BAP31 and survivin, we wondered how BAP31 and survivin regulated the chemosensitivity of HCC cells. As shown in Figure 5A, Western blot results showed that survivin silencing significantly increased Bax and reduced Bcl-2. Meanwhile, survivin silencing enhanced the effect of BAP31 knockdown in Dox-induced apoptosis. Conversely, we found that exogenous survivin overexpression increased Bcl-2 and reduced Bax expression, which rescued the effect of BAP31 knockdown in Dox-induced apoptosis of HepG2 and HuH-7 cells (Figure S4A). Together, these data supported that survivin is a downstream mediator of BAP31 in Dox-induced apoptosis. Next, flow cytometry results showed that knockdown of survivin or BAP31 significantly increased the number of apoptosis cells, and knockdown of both BAP31 and survivin further increased the apoptosis cells in HepG2 and HuH-7 cells (Figure 5B). A TUNEL assay was also performed to validate the results (Figure S4B).

Here, we hypothesized that knockdown of BAP31 and survivin has a synergistic effect on chemosensitivity by enhancing the apoptosis of HCC cells. MTT assay demonstrated that knockdown of survivin or BAP31 reduced the IC_50_ value, and knockdown of BAP31 and survivin further reduced the IC_50_ value of HepG2 and HuH-7 cells treated with Dox (Figure 5C). Meanwhile, the colony formation assay revealed that knockdown of survivin or BAP31 reduced cell colony formation ability. Furthermore, we found that the combination of knockdown of BAP31 and survivin resulted in a marked reduction in the colony formation ability of HepG2 and HuH-7 cells compared with knockdown of BAP31 alone (Figure S4C). Overall, these results indicated that knockdown of BAP31 and survivin has a synergistic effect on chemosensitivity by enhancing the apoptosis of HCC cells.

### 2.6. Knockdown of BAP31 Enhances the Antitumor Effect of Dox In Vivo

**Figure 6:**
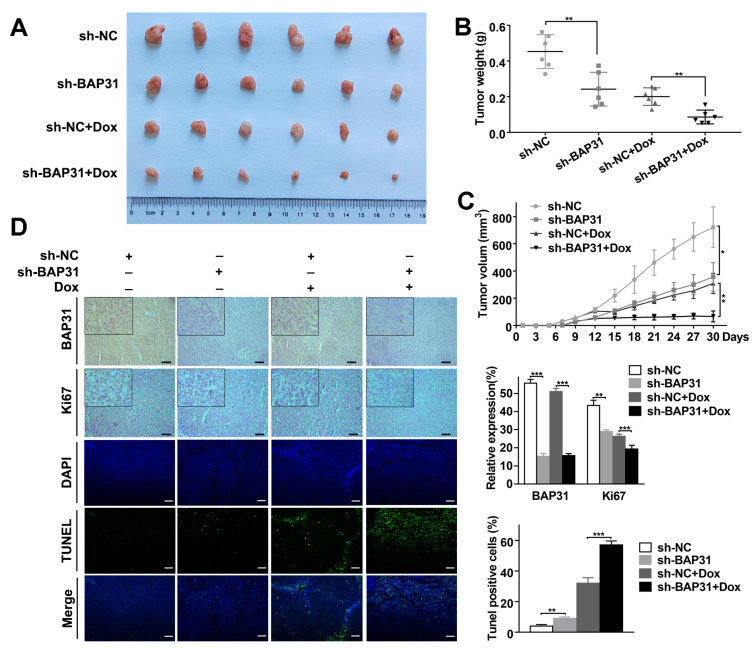
Knockdown of BAP31 enhances the antitumor effect of Dox in vivo. Mice were sacrificed 30 days after the indicated treatments, and the tumor volume (**A**) and weight (**B**) were measured. (**C**) Tumor size was monitored every three days in the different treatment groups. (**D**) The expression of BAP31 and Ki67 in tumor tissues of the different treatment groups as measured by immunohistochemistry (scale bars: 50 µm); TdT-mediated dUTP nick end labeling (TUNEL) assay in tumor tissues of the different treatment groups (scale bars: 200 µm). TUNEL-positive cells (green). Nuclei were stained with DAPI (blue). Data are represented as the mean ± SD of three independent experiments. β-actin was used as the loading control. * *p* < 0.05, ** *p* < 0.01, *** *p* < 0.001.

Xenograft mouse models were established by subcutaneously injecting HuH-7 cells transfected with sh-NC or sh-BAP31 into BALB/c nude mice. Dox (3 mg/kg) was initiated when the tumor size reached 100 mm^3^ within two weeks. At the end of treatment, tumors were harvested from the mice and examined. As shown in Figure S5A, there was no significant difference in the body weight of mice between the groups. Meanwhile, we found that the average tumor volume and weight of the sh-NC xenografts were larger than those of the sh-BAP31 xenografts. Dox treatment resulted in reduced tumor volume and weight of two xenografts. However, the average tumor volume and weight of the Dox-treated sh-NC xenografts were significantly larger than those of the Dox-treated sh-BAP31 xenografts (Figure 6A–C). These results indicated that BAP31 suppressed tumor growth, and that knockdown of BAP31 led to tumors that were more sensitive to Dox. Immunohistochemistry (IHC) confirmed that the expression of BAP31 was inhibited in sh-BAP31 and sh-BAP31+Dox group xenografts. Accordingly, we examined the expression of the proliferation marker Ki67 in tumor sections by IHC staining and found that BAP31 knockdown resulted in more apoptotic cells upon Dox treatment (Figure 6D). Moreover, a TUNEL assay confirmed that BAP31 knockdown or BAP31 knockdown increased apoptosis, and BAP31 knockdown treated with Dox further significantly increased the numbers of apoptotic cells (Figure 6D). Finally, the expression of BAP31, survivin, Bcl-2, and Bax proteins was detected by Western blot analysis, which was consistent with our previous results in vitro (Figure S5B). Collectively, these results implied that knockdown of BAP31 suppresses tumor growth and significantly enhances the antitumor effect of Dox in vivo.

## 3. Discussion

Dox is an effective antineoplastic agent against a wide range of cancers, and the development of chemoresistance limits its use [30,31]. Therefore, it is critical to find new targets for reducing chemoresistance. BAP31 has been reported to be involved in numerous biological processes, including proliferation [15,17], invasion [17,20], and apoptosis [32]. The evasion of apoptosis plays a crucial role in cancer development, recurrence, and chemoresistance [23,33,34]. In the present study, we found that BAP31 was abundantly expressed in cancer cells, and knockdown of BAP31 enhanced the chemosensitivity of cancer cells to Dox in vitro and in vivo. In addition, knockdown of BAP31 reduced the Dox resistance of HCC/Dox cells. Mechanistically, we clarified that knockdown of BAP31 downregulated survivin expression by increasing nucleus–cytoplasm translocation of FoxO1, which promoted Dox-induced apoptosis. This study suggested that knockdown of BAP31 may be a strategy to improve the efficacy of Dox in HCC.

Previous studies have identified the driving role of BAP31 in cancer progression [35]. BAP31 is emerging as an attractive therapeutic target for cancer. For example, Sun et al. reported that BAP31 promotes proliferation, invasion, and metastasis of liver cancer cells [18]. Dang et al. reported that BAP31 regulates proliferation, migration, and invasion to promote cervical cancer progression [17]. However, there is no study focused on whether BAP31 is involved in chemoresistance. This study aims to investigate the biological function and molecular mechanism of BAP31 in regulating HCC chemoresistance. In this study, we discovered that BAP31 was strongly expressed in BRCA, COAD, GMB, and HCC cells. Furthermore, we found that knockdown of BAP31 increased the chemosensitivity of Dox to cancer cells. These results suggested that BAP31 had a similar function of BRCA, COAD, GMB, and HCC cells in response to Dox, and the expression of BAP31 could serve as a marker for sensitivity to chemotherapy in these cancers (Figure 1). It is noteworthy that we found that the expression of BAP31 was higher in HepG2 and HuH-7 cells compared to SMMC-7721 cells. Meanwhile, the IC_50_ values of the knockdown of BAP31 were 6.5-fold and 11.2-fold lower in HepG2 and HuH-7 cells, respectively, compared with control cells. Thus, we selected two HCC cell lines (HepG2 and HuH-7) for use in subsequent experiments. These trends were also observed in the xenograft mouse models. The xenograft mouse models data indicated that knockdown of BAP31 reduced tumor growth and enhanced the antitumor effect of Dox (Figure 6). In addition, we found that Dox-resistant HCC cells had higher BAP31 expression than their parental cells, and BAP31 played a role as an oncogene in the process of Dox resistance of HCC/Dox cells. Knockdown of BAP31 resulted in lower cell viability and lower clone formation rate and overcame resistance to Dox in HCC/Dox cells (Figure 2). In summary, our study demonstrated that the efficacy of Dox was improved when knockdown of BAP31 in HCC/Dox cells was performed, and the effect was similar to that observed when knockdown of BAP31 in HCC cells was performed, which established the relationship between BAP31 and chemoresistance.

Apoptosis is a common mechanism of drug-induced tumor cell death, and altered apoptosis pathways are some of the important mechanisms underlying the development of the chemoresistance of cancer cells [36,37]. Studies have shown that apoptosis signaling pathways influence chemosensitivity. For example, Sun et al. reported that FGL1 regulates acquired resistance to gefitinib by inhibiting apoptosis in non-small cell lung cancer [38]. BAP31 primarily resides in the endoplasmic reticulum, which functions as a chaperone for specific transmembrane proteins and as a regulator of apoptosis [17]. It has been reported that BAP31 can regulate the expression of specific anti-apoptotic or apoptotic factors, including Bcl-2 and Bax, in certain cell types [18,32]. In this study, we found that knockdown of BAP31 enhanced apoptosis and increased Dox-induced apoptosis in HCC cells. To clarify the potential mechanisms of BAP31 in mediating Dox-induced apoptosis, we measured the expression of proteins related to apoptosis in cancer cells. By analyzing the relationship between BAP31 and 84 types of tumor-associated antigens using a Proteome Profiler Human XL Oncology Array, we found that BAP31 could significantly affect the expression of survivin. It has also been shown that survivin/Bax/Bcl-2 signaling is involved in the apoptosis of cancers [29,39]. Given that BAP31 has been shown to play critical roles in apoptosis, we speculated that BAP31 regulates survivin to promote apoptosis. Our results showed that knockdown of BAP31 exerted an apoptotic effect on HCC cells by inhibiting the expression of the anti-apoptotic factors survivin and Bcl-2 and enhancing the expression of the apoptotic factor Bax (Figure 3). However, the elucidation of the exact pathway by which BAP31 controls apoptosis needs further investigation.

Furthermore, we explored the underlying mechanisms of BAP31 in regulating survivin. Survivin is an IAP family member protein that localizes in mitochondria and functions as an apoptosis inhibitor protein [23,34]. Meanwhile, previous studies indicated that survivin could protect cancer cells from cell death induced by chemotherapeutic drugs [27,39]. Therefore, we reasoned that knockdown of BAP31 may reduce the expression of survivin to regulate Dox-induced apoptosis. Our data further showed that knockdown of BAP31 downregulated both the protein and mRNA expressions of survivin. Our previous studies have shown that BAP31 deficiency upregulates transcription factor translocation and transcriptional activity [40]. According to recent reviews, FoxO1 is known to be a key negative regulator for survivin transcription, and the promoter region of survivin contains the binding sites for FoxO1 [41,42]. Our study showed that knockdown of BAP31 promoted FoxO1 nucleus–cytoplasm translocation to inhibit survivin expression (Figure 4). However, the impact of BAP31 on FoxO1 needs to be studied further.

Chemotherapy drugs such as Dox regulate survivin in various types of cancer cells. Such regulation was found to be critical in determining the cellular response to the drugs [43,44]. Thus, our findings confirm a regulatory relationship between BAP31 and survivin, and they provide evidence that survivin serves as a downstream mediator of BAP31 in Dox-induced apoptosis. Similarly, some studies have shown that the inhibition of survivin signaling promotes the apoptosis of cancer cells. Next, we hypothesized that knockdown of BAP31 and survivin has a synergistic effect on chemosensitivity by enhancing the apoptosis of HCC cells. In the current study, we found that knockdown of BAP31 clearly reduced the expression of survivin, which had a synergistic effect on chemosensitivity by enhancing the apoptosis of HCC cells (Figure 5). Based on these findings, we demonstrated that knockdown of BAP31 increased cell apoptosis via the FoxO1/survivin signaling pathway and led to HCC becoming more sensitive to Dox. The intrabody approach is a gene-based strategy that blocks or modulates the function of target molecules. Based on the above conclusions, we hypothesized that a targeted intrabody against BAP31 might specifically block the interaction of BAP31, which could induce apoptosis. Targeting BAP31 using gene knockdown strategies, or in combination with Dox, may be a novel therapeutic strategy for treating HCC.

**Figure 7:**
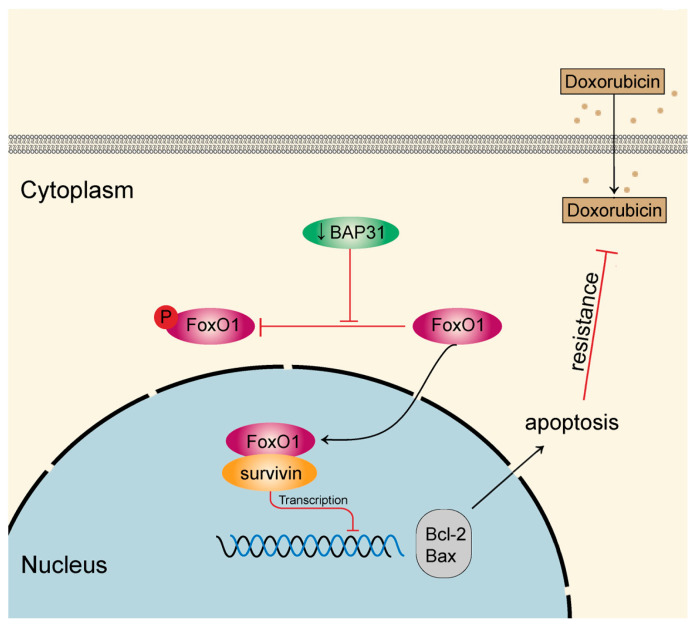
Schematic diagram. BAP31 knockdown promotes the nucleus–cytoplasm translocation of FoxO1, thus inhibiting the transcription of survivin. Knockdown of BAP31 increases the Dox-induced apoptosis and overcomes Dox resistance. (BAP31 knockdown is represented by ↓BAP31).

The findings of our study are summarized in a schematic diagram (Figure 7). Knockdown of BAP31 promotes FoxO1’s binding to the survivin promoter, thus inhibiting the transcription of survivin. Knockdown of BAP31 increases Dox-induced apoptosis and overcomes Dox resistance. The results of this study reveal a new strategy for downregulating BAP31, which may provide a breakthrough in overcoming Dox resistance.

## 4. Materials and Methods

### 4.1. Cell Culture and Chemicals

Two HCC cell lines (HepG2, HuH-7), a BRCA cell line (MCF-7), a COAD cell line (HCT116), and a GBM cell line (U251) were maintained in our laboratory. HepG2, MCF-7, HCT116, and U251 were cultured in DMEM medium (Gibco, New York, NY, USA), and HuH-7 was cultured in RPMI 1640 medium (Gibco) supplemented with fetal bovine serum, 100 U/mL penicillin, and 0.1 mg/mL streptomycin. Cells were routinely cultured in a humidified incubator at 37 °C under 5% CO_2_ atmosphere. Dox was purchased from Sigma-Aldrich/Merck KGaA (Darmstadt, Germany) at a purity of >95%.

### 4.2. Cell Viability Assay

Cell viability was examined by MTT assay. Cancer cells (2 × 10^3^ cells/well) were seeded in 96-well plates overnight in 5% CO_2_ at 37 °C. Cells were treated with Dox for 48 h. Next, cells were incubated with 5 mg/mL MTT reagents for another 4 h at 37 °C. After carefully removing the medium, dimethyl sulfoxide (200 μL) was added and agitated to dissolve the formazan crystals. The absorbance was recorded at 490 nm on a Synergy H1 microplate reader (Biotek, Burlington, VT, USA).

The long-term effect of Dox on cell proliferation was analyzed by colony formation assay. Cells were seeded in a 6-well plate. Next, cells were treated with Dox for 48 h and cultured in medium for 14 days. The medium was refreshed every two days. After that, cells were washed with phosphate buffer saline (PBS) three times, fixed with 4% paraformaldehyde, and stained with 0.1% crystal violet. Quantification of colony formation was performed using ImageJ software (V1.8.0).

### 4.3. Western Blot Analysis

Cells were lysed with radio immunoprecipitation assay buffer supplemented with phenylmethanesulfonyl fluoride (Beyotime, Shanghai, China) and quantified using the BCA protein concentration assay kit (Beyotime). Protein samples were separated by sodium dodecyl sulfate-polyacrylamide gel electrophoresis (Bio-Rad, Hercules, CA, USA) and electro-transferred to a polyvinylidene fluoride (0.22 μm). After blocking with 5% non-fat milk, the membranes were incubated with primary antibodies overnight at 4 °C. The next day, membranes were labeled with secondary antibodies. Signals were detected by Bio-Rad ChemiDoc™ imaging systems using an ECL detection kit (ThermoFisher Scientific, Waltham, MA, USA). β-actin was used as the endogenous control.

### 4.4. qRT-PCR Assay

RNA was extracted using Trizol (Sigma-Aldrich, Darmstadt, Germany) reagent as instructed by the protocol. cDNA synthesis was performed using a reverse transcription kit (Takara, Dalian, China) according to the manufacturer’s recommendations. Quantitative RT-PCR was conducted using 2× SYBR Green qPCR Master Mix (Vazyme, Nanjing, China), following the manufacturer’s protocol, on a CFX96 real-time PCR detection system, and GAPDH was used as a reference gene. The relative expression of the target genes was calculated using the 2-DDCt method. The primers (Sangon, Shanghai, China) were survivin-forward: 5′GAGGCTGGCTTCATCCACTG3′; survivin-reverse: 5′ATGCTCCTCTATCGGGTTGTC3′; GAPDH-forward: 5′GGAGCGAGATCCCTCCAAAAT3′; GAPDH-reverse: 5′GGCTGTTGTCATACTTCTCATGG3′; BAP31-forward: 5′CCTCTATGCGGAGGTCTTTGT3′; and BAP31-reverse: 5′CCGTCACATCATCATACTTCCGA3′.

### 4.5. Flow Cytometry Analysis

Flow cytometry analysis was used to detect cell apoptosis. In brief, 3 × 10^5^ cells were seeded in 6-well plates and transfected for 48 h, then treated with Dox for another 48 h. The treated cells were incubated with an annexin V-FITC/PI apoptosis detection kit (Meilunbio, Dalian, China) for cell apoptosis detection according to the manufacturer’s guidelines. Cells were harvested and analyzed using a flow cytometer (BD Biosciences, New York, NY, USA).

### 4.6. Immunofluorescence Analysis

Cells were seeded in 6-well plates for 48 h. Cells were fixed with 4% paraformaldehyde at 37 °C for 30 min. After fixation, cells were washed with PBS three times and incubated with 0.2% Triton X-100 in PBS for 30 min at room temperature. Then cells were blocked with 1% bovine serum albumin for 30 min at room temperature and stained with anti-BAP31 (1:250, Abcam, Cambridge, UK) and anti-FoxO1 (1:100, Wanleibio, Shenyang, China) for 12 h at 4 °C. After washing with PBS three times, cells were stained with the appropriate fluorescence-conjugated secondary antibody (CST, Danvers, MA, USA) for 1 h at room temperature and stained with DAPI (Beyotime). Cells were examined by fluorescence microscopy (Leica, Wetzlar, Germany).

### 4.7. Animal Experiments

5-week-old BALB/c nude mice were maintained in a specific-pathogen-free facility. Cells with indicated modification (5 × 10^6^ cells in 0.15 mL phosphate-buffered saline) were subcutaneously injected into the right flank of the BALB/c nude mice. Tumor volumes were measured every three days using calipers, and their volumes were calculated using the following formula: V = (A × B^2^)/2, where A and B are the maximal and minimal diameters in millimeters, respectively. Dox (3 mg/kg) was prepared in 0.9% NaCl and was injected intraperitoneally every two days for 16 days. A 0.9% NaCl solution served as the control. Mice were sacrificed at 30 days, after which the tumors were dissected and analyzed. There were no mice that died accidentally during the experiment, and the mice showed healthy vital signs.

### 4.8. IHC and TUNEL Assay

The resected tumor tissues were soaked in formalin and dehydrated, paraffin-embedded, and sectioned at 5 μm thickness. The sections were deparaffinized, hydrated, and microwaved for antigen removal. H_2_O_2_ (3%) was used to eliminate endogenous peroxidase activity. After incubation in 5% bovine serum albumin for 20 min to block non-specific binding, the sections were incubated with primary antibody at 4 °C overnight, followed by a biotinylated secondary antibody at 37 °C for 1 h. Then, the sections were stained with diaminobenzidine and counterstained with hematoxylin. Finally, all tissue sections were incubated in alcohol and xylene. The sections were observed under an inverted fluorescence microscope (Leica).

A TUNEL apoptosis assay kit (Wanleibio) was used to detect the apoptosis of HCC cells according to the manufacturer’s guidelines.

### 4.9. Statistical Analysis

All experimental data were analyzed using SPSS 22.0 statistical software, and the Student’s *t*-test was used to compare the means of two groups of independent samples. Data were presented as the mean SD of three independent experiments. The results with *p* < 0.05 were considered statistically significant.

## Data Availability

The data that support the findings of this study are available from the corresponding author upon reasonable request.

## Supplementary Materials

The following supporting information can be downloaded at: https://www.mdpi.com/article/10.3390/ijms24087622/s1.
