# Supplementary material for: Knockdown of BAP31 Overcomes Hepatocellular Carcinoma Doxorubicin Resistance through Downregulation of Survivin
Source: Int J Mol Sci. 2023 Apr 21;24(8):7622. doi: 10.3390/ijms24087622 (PMC10142662; doi:10.3390/ijms24087622)
Supplement: Supplementary file 1 [file ijms-24-07622-s001.zip › ijms-2318010-supplementary.pdf]

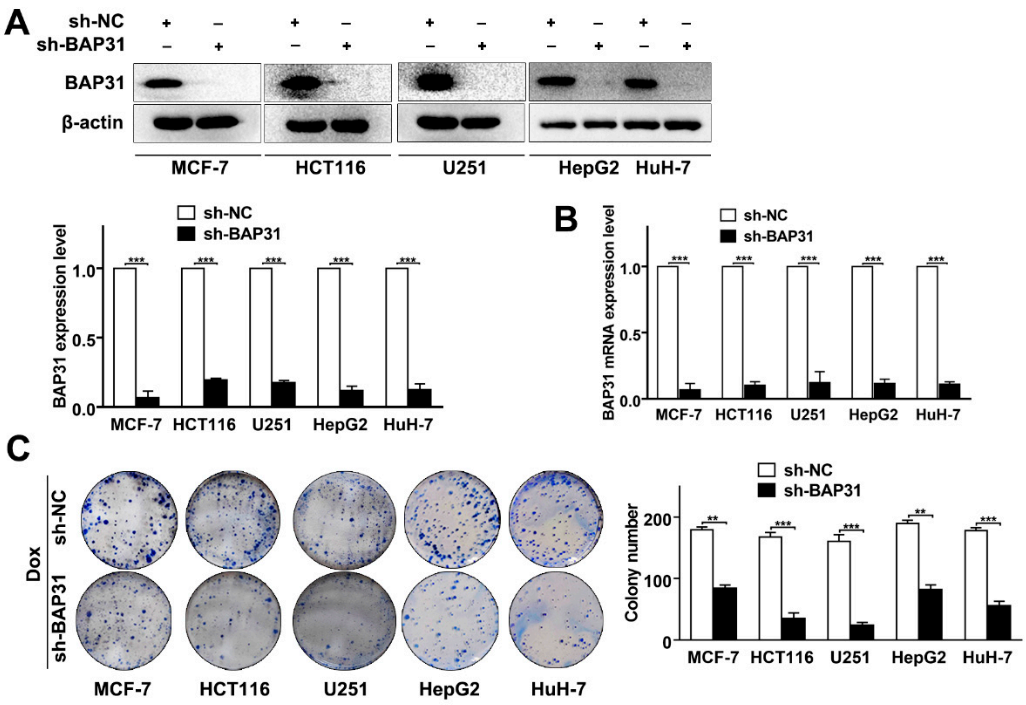

Figure S1. (**A**, **B**) Western blot and qRT-PCR were used to detect the expression of BAP31 in sh-NC and sh-BAP31 cells. (**C**) The colony formation assay was used to measure the colony formation ability of the indicated cells treated with Dox. Data are represented as the mean ± SD of three independent experiments. β-actin was used as the loading control. $^{**}p < 0.01$, $^{***}p < 0.001$.

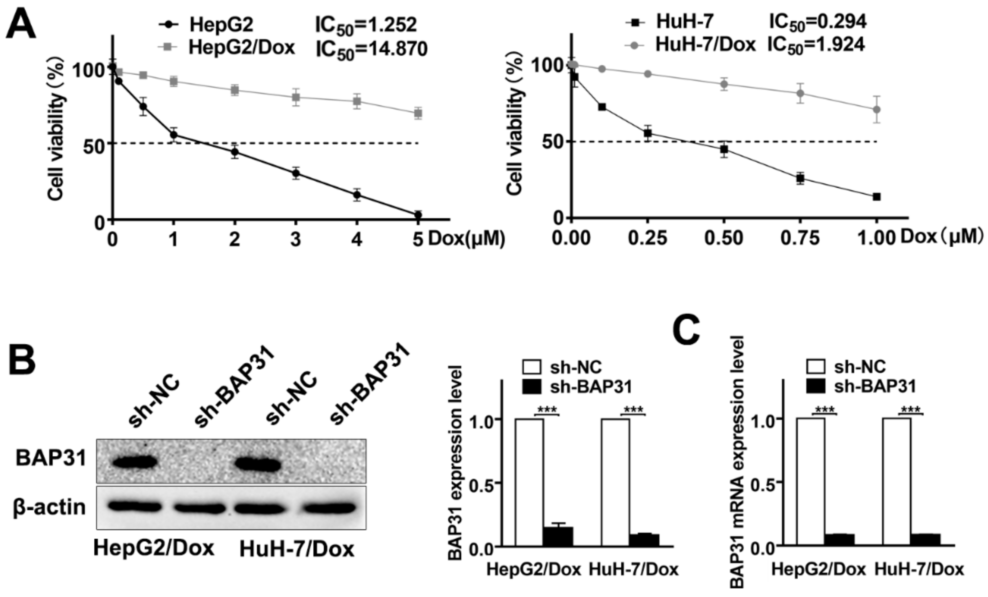

Figure S2. (**A**) IC_{50} values were determined by MTT assays in HCC and HCC/Dox cells treated with various concentrations of Dox. (**B, C**) Western blot and qRT-PCR were used to detect the expression of BAP31 in HCC and HCC/Dox cells. Data are represented as the mean ± SD of three independent experiments. β-actin was used as the loading control. $^{***}p < 0.001$.

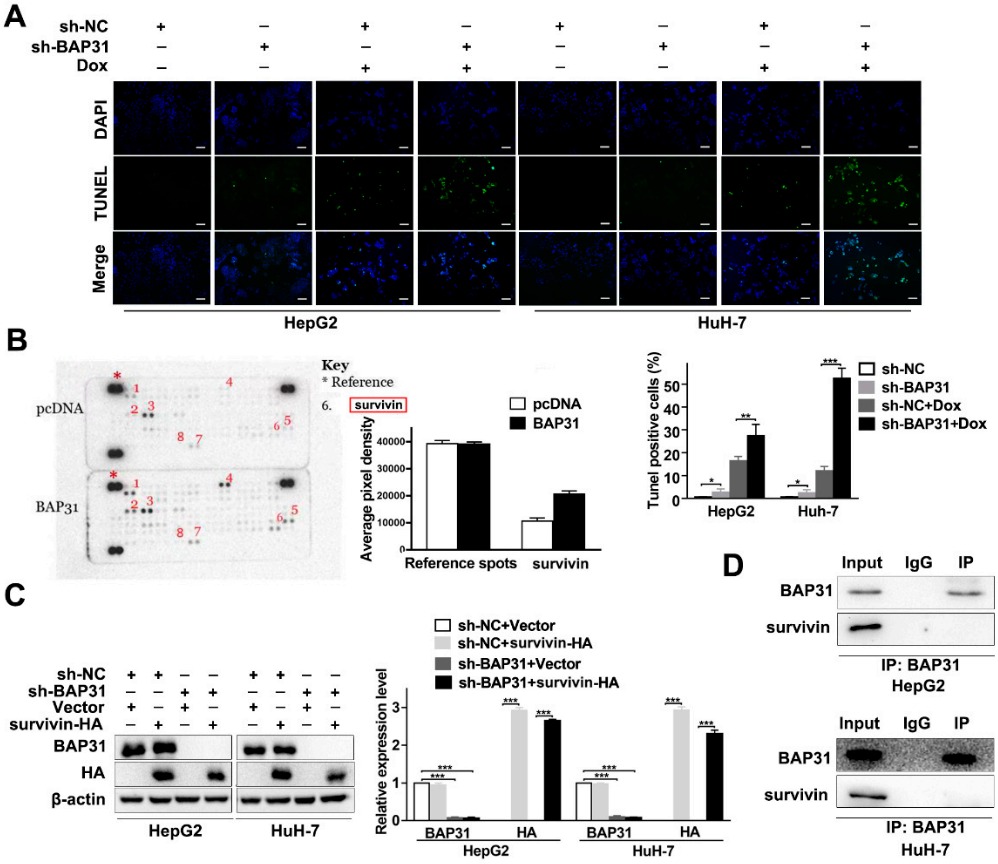

Figure S3. (**A**) TUNEL-positive cells (green) in the indicated cancer cells with or without Dox were observed under a fluorescence microscope (Scale bars: 200 μm). Nuclei were stained with DAPI (blue). (**B**) The Human XL Oncology Array detected multiple oncology-related proteins in the cell lysates. Reference Spots *. (**C**) Western blot and qRT-PCR were used to detect the expression of BAP31 and HA in indicated cancer cells transfected with or without survivin-HA. (**D**) Endogenous interaction between BAP31 and survivin was determined by immunoprecipitation with anti-BAP31 antibodies in HepG2 and HuH-7 cells. Data are represented as the mean ± SD of three independent experiments. β-actin was used as the loading control. *$p < 0.05$, **$p < 0.01$, ***$p < 0.001$.

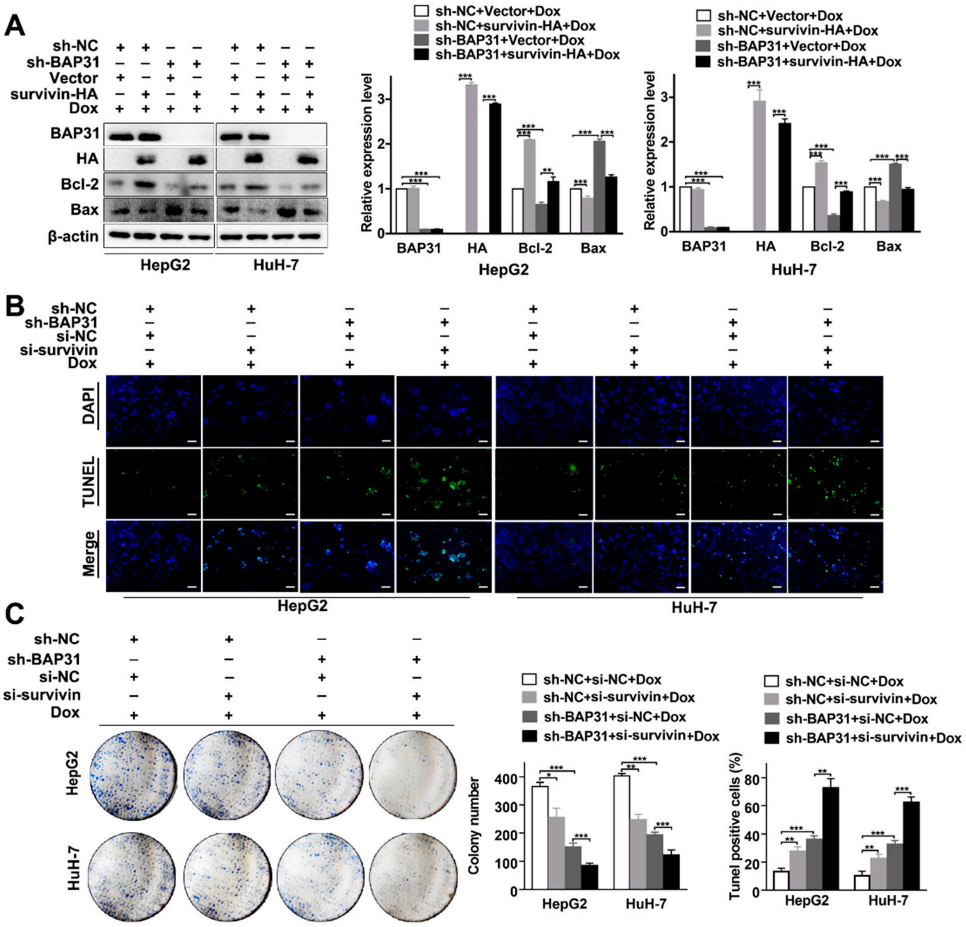

Figure S4. (**A**) Western blot was used to detect the expression of BAP31, HA, Bcl-2, and Bax in indicated cancer cells transfected with or without survivin-HA was treated with Dox. (**B**) TUNEL-positive cells (green) in indicated cancer cells transfected with or without si-survivin treated with Dox (Scale bars: 200 μm). Nuclei were stained with DAPI (blue). (**C**) The colony formation assay was used to measure the colony formation ability in indicated cancer cells transfected with or without si-survivin under the treatment with Dox. Data are represented as the mean ± SD of three independent experiments. β-actin was used as the loading control. $*p < 0.05$, $**p < 0.01$, $***p < 0.001$.

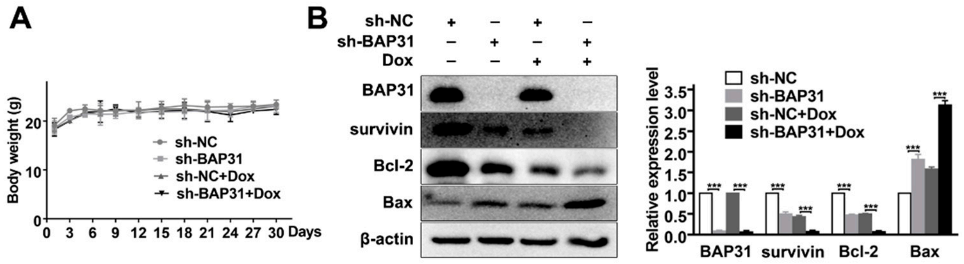

Figure S5. (**A**) The weight of mice was monitored every three days in the different treatment groups. (**B**) Western blot was used to detect the expression of proteins in tumor tissues of the different treatment groups. Data are represented as the mean ± SD of three independent experiments. β-actin was used as the loading control. $***p < 0.001$.
